# Association between Local Traffic-Generated Air Pollution and Preeclampsia and Preterm Delivery in the South Coast Air Basin of California

**DOI:** 10.1289/ehp.0800334

**Published:** 2009-06-23

**Authors:** Jun Wu, Cizao Ren, Ralph J. Delfino, Judith Chung, Michelle Wilhelm, Beate Ritz

**Affiliations:** 1 Program in Public Health, College of Health Sciences and; 2 Department of Epidemiology and; 3 Department of Obstetrics and Gynecology, School of Medicine, University of California, Irvine, California, USA; 4 Department of Epidemiology, School of Public Health, University of California, Los Angeles, California, USA

**Keywords:** air pollution, nitrogen oxides, particulate matter, preeclampsia, pregnancy outcome, preterm birth, vehicle emission

## Abstract

**Background:**

Preeclampsia is a major complication of pregnancy that can lead to substantial maternal and perinatal morbidity, mortality, and preterm birth. Increasing evidence suggests that air pollution adversely affects pregnancy outcomes. Yet few studies have examined how local traffic-generated emissions affect preeclampsia in addition to preterm birth.

**Objectives:**

We examined effects of residential exposure to local traffic-generated air pollution on preeclampsia and preterm delivery (PTD).

**Methods:**

We identified 81,186 singleton birth records from four hospitals (1997–2006) in Los Angeles and Orange Counties, California (USA). We used a line-source dispersion model (CALINE4) to estimate individual exposure to local traffic-generated nitrogen oxides (NO_x_) and particulate matter < 2.5 μm in aerodynamic diameter (PM_2.5_) across the entire pregnancy. We used logistic regression to estimate effects of air pollution exposures on preeclampsia, PTD (gestational age < 37 weeks), moderate PTD (MPTD; gestational age < 35 weeks), and very PTD (VPTD; gestational age < 30 weeks).

**Results:**

We observed elevated risks for preeclampsia and preterm birth from maternal exposure to local traffic-generated NO_x_ and PM_2.5_. The risk of preeclampsia increased 33% [odds ratio (OR) = 1.33; 95% confidence interval (CI), 1.18–1.49] and 42% (OR = 1.42; 95% CI, 1.26–1.59) for the highest NO_x_ and PM_2.5_ exposure quartiles, respectively. The risk of VPTD increased 128% (OR = 2.28; 95% CI, 2.15–2.42) and 81% (OR = 1.81; 95% CI, 1.71–1.92) for women in the highest NO_x_ and PM_2.5_ exposure quartiles, respectively.

**Conclusion:**

Exposure to local traffic-generated air pollution during pregnancy increases the risk of preeclampsia and preterm birth in Southern California women. These results provide further evidence that air pollution is associated with adverse reproductive outcomes.

Preeclampsia is a multisystem disorder in pregnant women, which is characterized by elevated blood pressure, edema, and protein in the urine. Preeclampsia complicates an estimated 2–8% of pregnancies and is a major cause of maternal mortality and morbidities, perinatal deaths, preterm birth, and intrauterine growth restriction (Duley 1992; Sibai et al. 2005). Because the only cure is delivery of the baby and placenta, preeclampsia is the most frequent primary reason for elective nonspontaneous preterm birth, accounting for 30–35% of total preterm deliveries (PTD) (Goldenberg et al. 2008; Meis et al. 1998). Preeclampsia does not necessarily lead to spontaneous PTD, and the association between preeclampsia and spontaneous PTD depends on PTD subtypes defined by gestational age (e.g., very or moderately preterm) and pathway (e.g., membrane rupture or spontaneous onset of labor before membrane rupture) (Ananth et al. 1997).

More than half a million infants are born prematurely each year in the United States (Hamilton et al. 2006). Preterm birth is associated with 70% of neonatal deaths and up to 75% of neonatal morbidity (Challis et al. 2001). Extremely preterm infants who survive the neonatal period face an elevated risk of serious life-long health problems, including learning disabilities and other chronic conditions (Doyle 1995, 2008). A growing body of research has linked elevated air pollutant exposures to PTD at pollution levels typical of many U.S. cities (Maroziene and Grazuleviciene 2002; Perera et al. 2003; Ritz et al. 2000, 2007; Šrám et al. 2005; Wilhelm and Ritz 2005). So far, preeclampsia has been associated with air pollution in only two recent U.S. studies (Rudra and Williams 2006; Woodruff et al. 2008).

There is also a growing body of evidence linking pollutants found in traffic exhaust specifically to respiratory and cardiovascular diseases (Adar and Kaufman 2007; Delfino 2002; Heinrich and Wichmann 2004; Sarnat and Holguin 2007). Although data are limited to date (de Kok et al. 2006), evidence is emerging that fresh vehicle emissions contain more toxic compounds per unit of particle mass than do aged aerosols, in part because of the contribution of ultrafine particles (UFPs; < 0.1 μm in aerodynamic diameter), which are found in higher concentration closer to emission sources (Zhu et al. 2002). Fresh traffic emissions’ toxicity may originate from a high concentration of organic components because particle number concentrations are orders of magnitude higher, increasing the surface area to which volatile and semivolatile pollutants such as polycyclic aromatic hydrocarbons (PAHs) and carbonyl compounds can adhere.

There is indirect evidence for adverse impacts of traffic-generated PAHs on birth outcomes from studies in the United States (Choi et al. 2006, 2008; Perera et al. 2003, 2004), Poland (Choi et al. 2006), and the Czech Republic (Dejmek et al. 2000). However, most previous birth outcome studies relied solely on data from air monitors operated by government agencies, which are usually sited to assess regional ambient pollution and are thus unlikely to adequately capture the high spatial heterogeneity of air pollutants directly emitted from traffic (Hitchins et al. 2000; Zhu et al. 2002). Two studies examined the impact of local traffic emissions specifically on PTD (Wilhelm and Ritz 2003; Yang et al. 2003), but both assigned exposures based on the distance to and/or level of traffic on major roadways near residences, a relatively crude measure of traffic exhaust that does not consider vehicle emission rates or meteorology (Jerrett et al. 2005). Two recent birth outcome studies (Brauer et al. 2008; Slama et al. 2007), however, employed more sophisticated techniques to model traffic-related air pollution based on land use regression (LUR) that yielded quantitative estimates for specific pollutants.

Because of population and economic growth and the lack of effective public transportation in the Los Angeles area, the amount of passenger traffic and of goods being moved through this region is projected to increase substantially in the next decade (California Environmental Protection Agency 2006). Such rapid growth in traffic-related fossil fuel use is expected to occur worldwide (Energy Information Administration 2008), adding urgency to research evaluating the impact of increased vehicle emissions on reproductive health outcomes. To address this issue, in the present study we investigated the effects of local traffic-generated air pollution on preterm birth and preeclampsia based on the CALINE4 line-source dispersion model (Benson 1989), which is specifically designed for the assessment of traffic emissions from roads. We obtained health outcomes data from a perinatal database with detailed clinical data from a four-hospital network in southern Los Angeles County and Orange County, California, from 1997 to 2006.

## Materials and Methods

### Study population

Our racially and socio-economically diverse study population resided in two areas of the South Coast Air Basin of California [see Supplemental Material, Figure 1 (doi:10.1289/ehp.0800334.S1 via http://dx.doi.org)] that exhibit a wide range of air pollution concentrations from mobile sources. One is located in southern Los Angeles County, north of the Ports of Los Angeles and Long Beach. The communities adjacent to the port are affected by major commuter freeways and main trucking routes for goods leading out of the port (Interstate 710); 15% of all containers arriving in the United States travel on this freeway (Beverly 2005). The other area is located in Orange County, southeast of the ports. Four major commuter and truck transport freeways traverse these neighborhoods. The study region also covers some suburban, low-traffic neighborhoods.

### Birth data

We acquired all birth-related variables and antenatal information for pregnant women delivering during 1997–2006 within the Memorial Health Care System (MHCS), a four-hospital network (Chung et al. 2006). Poverty (percentage of population living below the poverty level) information by census block groups was obtained from U.S. Census 2000 data (U.S. Census Bureau 2004). No birth certificate data were used. The MHCS database included residential address at delivery, birth hospital, prenatal care insurance, maternal age and race/ethnicity, maternal medical history (heart disease, chronic hypertension, previous PTD), preeclampsia and other maternal complications during pregnancy (diabetes, pyelonephritis), parity (first birth vs. second or subsequent birth), gestational age, and the neonate’s sex and birth weight. Gestational age was calculated according to delivery date and estimated date of conception (based on last menstrual period and ultrasound dating). We did not have diagnosis dates for preeclampsia; therefore, we could not determine when the disease first occurred.

A total of 105,092 neonatal records were extracted from the birth database. We successfully geocoded 92.8% of nonmissing residential addresses with exact matches to house number using the TeleAtlas Geocoding Service (http://www.geocode.com). A total of 81,186 singleton birth records remained in the data set for final analyses (77%) after excluding multiple gestations (*n* = 5,261; 5%), incomplete records including those without full residential address and those missing any covariate information (*n* = 12,666; 12%), and unsuccessfully geocoded residential addresses and addresses outside the study region (*n* = 5,979; 6%). Excluded births were similar to included births by study region (Los Angeles County and Orange County), demographics (age and race/ethnicity), and the prevalence of preeclampsia.

### Air pollution exposure assessment

Background air pollutant concentrations may be high in the study region due to port activities and relatively heavy traffic regionwide. However, in this study we focused solely on local traffic-generated pollution to assess the potentially high toxicity of hypothesized causative agents (e.g., UFPs and PAHs) in traffic emissions. Our estimated pollutant exposures should be regarded as indicators of primary emissions from local vehicular traffic on top of background ambient levels [see Supplemental Material (doi:10.1289/ehp.0800334.S1)]. We modeled local traffic pollution using a modified CALINE4 dispersion model for two surrogate pollutants [nitrogen oxides (NO_x_) and particulate matter ≤ 2.5 μm in aerodynamic diameter (PM_2.5_)] originating from traffic emissions within 3 km of each residence (Benson 1989; Wu et al. 2005, 2009), assuming that at this distance we would capture most local traffic emissions but little regional pollution transported from upwind areas. CALINE4 is a Gaussian dispersion model that employs a mixing zone concept to characterize pollutant dispersion over the roadway. Major inputs to CALINE4 include meteorology (atmospheric stability, mixing height, wind, and temperature), roadway geometry and traffic activities, and vehicle emission factors. The performance of CALINE4 has been evaluated in a number of studies (Benson 1989, 1992; Broderick et al. 2005; Gramotnev et al. 2003; Levitin et al. 2005; Marmur and Mamane 2003). Previous studies have found moderate to high correlations (*R* = 0.55–0.95) of CALINE4-modeled estimates with measured variability of traffic-related air pollutants [e.g., NO_x_ and nitrogen dioxide] in urban communities (Gauderman et al. 2005; Jerrett 2006). Our recent study showed a high correlation (*R* = 0.87) of CALINE4-modeled monthly NO_x_ concentrations with measurements at nine monitoring sites in the Long Beach study area in December 2007 and April 2008 (Wu J, Lurmann F, Avol E, unpublished data).

A comprehensive traffic database with annual average daily traffic counts and gasoline and diesel vehicle fractions was constructed for the entire study region. Vehicle emission factors were obtained from the California Air Resources Board’s EMFAC2007 vehicle emissions model (California Air Resources Board 2008). Paved road-dust emissions for PM_2.5_ were based on in-roadway measurements (Fitz and Bufalino 2002). Hourly wind speed, direction, and temperature were obtained from the National Weather Service (National Climatic Data Center 2008). Summarized mixing heights by season and hour were obtained from the 1997 Southern California Ozone Study (Croes and Fujita 2003) and assigned to each modeled day based on season and hour.

### Statistical analyses

PTD was defined as a birth at < 37 completed gestational weeks, moderate preterm deliveries (MPTD) as births at < 35 gestational weeks, and very preterm deliveries (VPTD) as births at < 30 gestational weeks. We defined preeclampsia as the occurrence of mild preeclampsia (blood pressure > 140/90 mmHg and proteinuria), severe preeclampsia (e.g., blood pressure > 160/110 mmHg and proteinuria with or without signs of end-organ involvement, including oliguria, liver function abnormalities, thrombocytopenia, headache), or hemolysis, elevated liver enzyme levels, and low platelet count (HELLP) syndrome at any time during pregnancy. Because hemolysis/HELLP is on the continuum of mild/severe preeclampsia and is relatively uncommon, we chose to combine this diagnosis with severe preeclampsia. Pregnancy trimesters were defined as gestational weeks 1–13, 14–26, and 27 weeks to birth.

We performed multiple logistic regression using the statistical package R (version 2.6.1; R Foundation for Statistical Computing, Vienna, Austria). Confounders were selected based on *a priori* knowledge and included maternal age, maternal race/ethnicity, parity, prenatal care insurance type [private, public (government-sponsored or self-pay), and unknown], poverty, season of conception, pyelonephritis (preterm analyses only), and diabetes (preeclampsia analyses only). We adjusted for maternal age as a continuous variable using a quadratic polynomial function. For the preeclampsia analyses, we excluded women who had preexisting chronic conditions such as hypertension and heart disease before pregnancy. We separately calculated odds ratios (ORs) and 95% confidence intervals (CIs) for increases in the interquartile range (IQR) for each pollutant exposure metric. ORs and 95% CIs were scaled to IQR increases in air pollutant variables to standardize and compare associations regardless of pollutant concentration range or units of measurement (Lipfert and Wyzga 1999). In addition to using continuous exposure variables, we performed categorical analyses in which we compared subjects in each exposure quartile with those in the lowest quartile and tested for dose response. We also examined the outcomes both collectively and separately by subcategories, including study region, race, poverty, insurance type, infant sex, maternal age, parity, delivery type and method, and health conditions (diabetes for preeclampsia and preeclampsia for preterm birth).

## Results

### Descriptive statistics

Most mothers were non-Hispanic white or Hispanic (Table 1). The prevalence of preeclampsia was higher among PTD women compared with non-PTD women (12% vs. 2%) and among African-American women compared with other races (4% vs. 3%). Mild, severe, and HELLP syndrome accounted for 75%, 18%, and 7% of the preeclampsia cases, respectively. The prevalence of PTD was higher among male infants than among female infants (9% vs. 8%) and in African-American women than in other races (13% vs. 7–9%). Spontaneous delivery accounted for 79% of all births and occurred in 87% of preterm births and 78% of term births. The poverty rate in our study region was higher than the national average (14% vs. 11% based on 2000 Census data) (U.S. Census Bureau 2004).

Average air pollution exposures derived from the CALINE4 model for each pregnancy period and during the entire pregnancy were similar and moderately to strongly correlated (Table 2). CALINE4-estimated average monthly (over all subjects in each calendar month) NO_x_ exposures showed a clear seasonal trend, with higher exposures in the cool season (average of 10.8 ppb in December) and lower exposures in the warm season (average of 5.8 ppb in June), and we observed a very similar monthly trend for PM_2.5_. The estimated concentrations were much lower than those measured at three ambient monitoring stations in the area (e.g., annual mean of 57.0 ppb NO_x_), likely because our model estimates were for local traffic-generated emissions only. As expected, the modeled NO_x_ and PM_2.5_ exposures were highly correlated (correlation coefficient *r* = 0.91) in every pregnancy trimester, because the two pollutants are emitted by the same source: local traffic.

### Regression analyses

Because of only slight variations in exposures and effect estimates in different pregnancy periods [see Supplemental Material, Tables 1 and 2 (doi:10.1289/ehp.0800334.S1)], we present all regression results based on exposure during the entire pregnancy period. We found positive associations of preeclampsia and preterm birth with entire-pregnancy exposure to traffic-related air pollution (Table 3). An 11% increase was observed in adjusted risk of preeclampsia per IQR increase of entire-pregnancy NO_x_. Preeclampsia results were the same for modeled PM_2.5_ exposures. Overall, we observed somewhat stronger increases in risk of preterm birth with increases in modeled NO_x_ than with modeled PM_2.5_. The effect of exposure tended to be stronger for VPTD (25% increase in risk per IQR increase in NO_x_) than for PTD considered as a whole (6% increase in risk per IQR increase in NO_x_).

Stratified analyses for preeclampsia and preterm birth were conducted [see Supplemental Material, Tables 3–6 (doi:10.1289/ehp.0800334.S1)]. We found greater impacts of traffic-related air pollution on preeclampsia and VPTD for women ≥ 40 years of age and in women < 20 years of age when giving birth, although 95% CIs overlapped to a large degree. We observed a higher risk of preeclampsia from local traffic-generated air pollution exposure among privately insured women than among women on public or government-sponsored insurance [for entire-pregnancy NO_x_: interquartile OR (IOR) = 1.12; 95% CI, 1.06–1.18, vs. IOR = 1.04; 95% CI, 0.96–1.13]. Closer inspection, however, showed that this was mostly driven by the high percentage (83%) of older women (> 40 years of age) using private insurance (for > 40 age group: IOR = 1.44; 95% CI, 1.22–1.69; ≤ 40 age group: IOR = 1.09; 95% CI, 1.03–1.16; based on entire-pregnancy NO_x_) versus public or government-sponsored insurance (for > 40 age group: IOR = 0.93; 95% CI, 0.56–1.54; for ≤ 40 age group: IOR = 1.05; 95% CI, 0.97–1.14; based on entire-pregnancy NO_x_). We observed no significant differences in effect estimates by study region, race/ethnicity, poverty, infant sex, parity, delivery type (spontaneous vs. nonspontaneous), delivery method (vaginal vs. cesarean section), diabetes status (for preeclampsia), and preeclampsia (for preterm birth).

**Figure 1:**
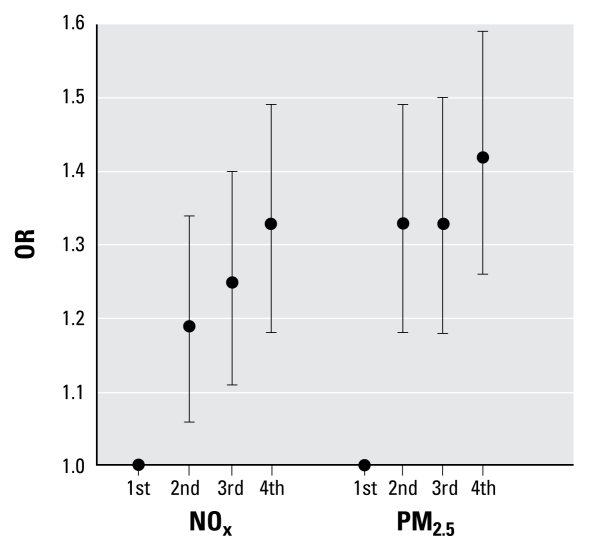
Adjusted ORs (95% CIs) for preeclampsia by entire-pregnancy exposure quartile (adjusted for maternal age, maternal race/ethnicity, parity, prenatal care insurance type, poverty, diabetes, and season of conception).

**Figure 2:**
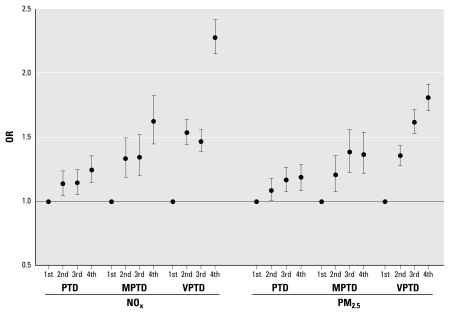
Adjusted ORs (95% CIs) for PTD, MPTD, and VPTD by entire-pregnancy exposure quartile (adjusted for maternal age, maternal race/ethnicity, parity, prenatal care insurance type, poverty, pyelonephritis, and season of conception).

Preeclampsia risk increased with quartiles of modeled NO_x_ and PM_2.5_ exposures, and the increase was consistent with a linear dose response for NO_x_ (Figure 1). We observed a 33% (OR = 1.33; 95% CI, 1.18–1.49) and 42% (OR = 1.42; 95% CI, 1.26–1.59) increase in risk of preeclampsia for women in the highest NO_x_ and PM_2.5_ entire-pregnancy exposure quartiles, respectively. We observed increasing risks with increasing quartiles of exposure to modeled NO_x_ and PM_2.5_ and all preterm birth outcomes, yet the pattern was not always linear with dose (Figure 2). We observed a 128% (OR = 2.28; 95% CI, 2.15–2.42) and 81% (OR = 1.81; 95% CI, 1.71–1.92) increase in risk of VPTD for women in the highest NO_x_ and PM_2.5_ entire-pregnancy exposure quartiles, respectively. The dose–response relationships from the quartile categorical analyses were consistent with what we observed from smoothing curves of dose response [see Supplemental Material, Figure 2 (doi:10.1289/ehp.0800334.S1)].

## Discussion

There is growing interest in exploring the possible effects of ambient air pollution on fetal and perinatal development because the growing fetus may be particularly susceptible to the toxic effects of air pollutants (Maisonet et al. 2004; Mone et al. 2004; Pinkerton and Joad 2006). Our study contributes new results based on exposure data from a dispersion model for local traffic-generated air pollutants and preeclampsia. To our knowledge, this is the first study to show a positive association between exposure to local traffic-generated pollutants at the birth residence and the development of preeclampsia during pregnancy. We also found that the risk of premature birth increases with exposure to local traffic-generated pollutants, and this risk was strongest for VPTD followed by MPTD and PTD. This is important because postnatal health impairments are greatest for the children born most premature (Doyle 1995, 2008).

The present study had two major advantages over previous studies examining traffic air pollution and birth outcomes. First, we modeled air pollution exposures from local traffic sources (within 3 km) using a comprehensive traffic database and a well-established dispersion model that better characterizes spatiotemporal variability in exposure than that used in most previous studies. Two exceptions are recent studies from Munich, Germany (Slama et al. 2007), and Vancouver, Canada (Brauer et al. 2008), that employed temporally adjusted LUR models. However, because the LUR models were based on ambient air measurement data, they estimated total ambient air pollutant concentrations with contributions from many sources other than local traffic emissions. Thus, these exposure estimates only partially represent local traffic-generated air pollutants. The amount of total estimated ambient pollution that is contributed from traffic may vary according to location in LUR models. In addition, LUR models may not perform well in predicting temporal variations of exposures because they are mostly built relying on one to four purpose-designed monitoring windows of 7 to 14 days, with or without further temporal adjustment using ambient monitoring station data (Hoek et al. 2008).

The second major advantage is that the present study used detailed individual-level clinical data (e.g., chronic hypertension, pyelonephritis, diabetes, heart disease), allowing us to evaluate the impact of these clinical parameters on air pollution effect estimates. But more important, we were able to employ more accurate gestational age information to classify preterm birth than most previous air pollution studies that relied on birth certificates. Gestational ages on birth certificates are usually based on first day of last menstrual period, which leads to misclassification of gestational age due to poor recall, postconception bleeding, or menstrual irregularities (Dietz et al. 2007; Kline 1989; Lynch and Zhang 2007). One the other hand, gestational age estimated by ultrasound measurements alone may induce systematic errors and inflate the risk of PTD (Olsen and Basso 2005). More than 99% of our subjects obtained prenatal care early in pregnancy, which ensured that their estimated conception date was based on a combination of last menstrual period and early ultrasound dating. Moreover, our preeclampsia data were based on hospital records of clinical diagnoses, probably more accurate than the preeclampsia data reported on birth certificates that may only record extreme or severe preeclampsia cases.

We estimated only local traffic-generated air pollution exposure in this study, whereas most previous studies based on ambient or modeled total concentration data (e.g., carbon monoxide and NO_x_) examined contributions from not only local traffic but also pollutants transported from upwind regions and from other sources. Local traffic emissions may differ from aged pollutants from long-range transport in terms of chemical composition and particle size distribution. Therefore, our estimated effect sizes for different outcomes may not be directly comparable to those from other air pollution studies.

The only results ever reported for air pollution and preeclampsia relied on CO concentrations measured at the nearest ambient air monitor to residence (Woodruff et al. 2008) or CO and PM_2.5_ concentrations estimated using linear regression models at each residence (Rudra and Williams 2006). Rudra and Williams (2006) observed a 49% increase in preeclampsia risk (95% CI, 0.76–2.90) for third- versus first-tertile average CO exposures during the month of conception and the following 3 months among women in Seattle, Washington, and Woodruff et al. (2008) reported an 8% increase in preeclampsia risk (95% CI, 1.02–1.14) for the highest versus the lowest entire-pregnancy CO exposure quartile in Californian women. In our study, women exposed at the highest quartile of modeled entire-pregnancy PM_2.5_ experienced approximately 40% higher risk of developing preeclampsia compared with women in the lowest quartile of exposure. We also noted a slightly higher risk of preeclampsia from local traffic-generated air pollution exposure among privately insured women. This might have been attributable to the high percentage (83%) of older women (> 40 years of age) using private insurance, as mentioned above, or may result from more accurate diagnosis of preeclampsia in privately compared with publicly insured women. Also, older pregnant women might be especially vulnerable to the effects of toxins such as air pollutants.

Our preterm birth results are consistent with results from previous birth outcome studies in the literature. In addition, the preterm birth results were similar using separate models for the three nonexclusive preterm outcomes compared with multilogit models that captured the relatedness of the three outcomes [see Supplemental Material, Table 7 (doi:10.1289/ehp.0800334.S1)]. We estimated a 6% increase in risk of PTD per IQR in modeled entire-pregnancy NO_x_ exposure and a 25% increased risk of PTD for mothers in the highest NO_x_ exposure quartile. Wilhelm and Ritz (2003) previously reported a 10–20% increase in the risk of PTD in mothers exposed to high levels of local traffic-generated air pollution in Southern California, based solely on residential distance-weighted traffic density. Following up on this first study, they conducted a nested case–control study within another birth cohort in Los Angeles County, California, and found PTD to be approximately 20% higher in mothers with first trimester CO exposure > 1.25 ppm (Ritz et al. 2007). A study from Taiwan estimated a 30% increased risk of PTD for mothers living within 500 m of a major freeway (Yang et al. 2003). A more recent study from Vancouver, Canada, reported no consistent association of PTD (< 37 weeks) with any of the pregnancy air pollution exposure metrics (including LUR measures) except inverse distance–weighted PM_2.5_ concentration during the entire pregnancy (OR = 1.06; 95% CI, 1.01–1.11; per 1-μg/m^3^ increase in PM_2.5_) (Brauer et al. 2008). Similar to our findings, risk increased when they further restricted PTD to < 30 weeks of gestation (for PM_2.5_ exposure: OR = 1.13; 95% CI, 0.92–1.39; for NO_x_ exposure: OR = 1.26; 95% CI, 1.08–1.47).

We found the risk of preeclampsia and VPTD due to modeled NO_x_ and PM_2.5_ exposure from traffic to be greater in the youngest (< 20 years of age) and the oldest (≥ 40 years of age) age groups, consistent with the pre-term birth results of a study in Los Angeles County, California (Ponce et al. 2005). Two U.S. studies, one conducted in Arizona and North Dakota (Ahluwalia et al. 1997) and the other in California (Windham et al. 2000), have also reported a stronger impact of environmental tobacco smoke (ETS) on preterm births among older (≥ 30 years of age) compared with younger (< 30 years of age) mothers, further suggesting possible differences in vulnerability by maternal age.

There were several limitations in the present study. We likely reduced exposure measurement error for primary traffic pollutants by using a dispersion model and a sophisticated traffic database versus relying on ambient measurements. However, the exposure estimates were based solely on the maternal address at time of birth. Mobility rates among pregnant women reported in the literature range from 12% (Fell et al. 2004) to 35% (Brauer et al. 2008). Ritz et al. (2007) found that associations between air pollution exposures (estimated via nearest air monitor) during pregnancy and preterm birth did not change or slightly strengthened when restricting analyses to women who did not move during pregnancy. The estimates of exposures in the present study, however, may have been affected more strongly by residential mobility because they are more spatially resolved than in previous studies. Second, our exposure estimates were based only on residential addresses, ignoring other microenvironments (e.g., workplace, commuting) that might be important for personal exposures. Ritz et al. (2007) reported associations between monitor-based estimates of air pollution exposure during pregnancy and PTD to be greater for women who did not work (and for whom a residence-based measure of exposure presumably is more accurate) than for women who worked outside their homes.

Another potential source of bias is residual confounding due to risk factors we were unable to account for in our analyses (e.g., maternal smoking, ETS, stress, and nutrition). Ritz et al. (2007) collected detailed survey data postnatally on risk factors not reported on birth certificates and assessed the influence of these potential confounding factors on air pollution effect estimates for preterm birth. Adjustment for covariates on birth certificates exhibited the strongest influence on the pollutant effect estimates, whereas additional adjustment for a large number of survey covariates (e.g., occupation, income, maternal smoking and ETS, alcohol drinking) changed the effect estimates by < 5%. This confirmed that for pollutants that change with season and are averaged over short time intervals (pregnancy months or trimesters), behavioral factors that do not change seasonally are unlikely to be confounders. Compared with ambient measurements of total pollutant concentrations, however, the major contrast in the CALINE4-modeled exposure was spatial rather than temporal. Therefore, residual confounding cannot be ruled out in these primarily spatially based exposure measures.

It is also uncertain to what degree the dispersion model we used represents pollutant species released only by traffic. Comparing modeled and measured concentrations, we observed reasonable agreement between CALINE4-modeled and measured 2-week average NO_2_ concentrations at 260 residences in six communities participating in the Southern California Children’s Health Study, with an *R*^2^ ranging from 0.3 to 0.9 (Gauderman et al. 2005; Jerrett 2006). Relatively high correlations (*n* = 14; *R*^2^ = 0.76) were found between CALINE4-modeled and measured monthly average concentrations of NO_x_ at nine monitoring stations in the Long Beach area in November 2007 and/or April 2008 (Wu J, Lurmann F, Avol E, unpublished data). The *R*^2^ for daily estimates ranged from 0.19 to 0.81 (mean = 0.36) among the nine stations (Wu J, Lurmann F, Avol E, unpublished data). Thus, we expect that longer-term exposure estimates (monthly, trimester, and entire-pregnancy averages) derived from CALINE models closely reflect residential exposure to local traffic-generated pollutants because traffic counts and mixing heights are based on long-term, annual, or seasonal average observations.

Both modeled PM_2.5_ and NO_x_ were associated with PTD and preeclampsia, but this should not be interpreted to mean that these pollutants are necessarily causative for these adverse outcomes; rather, they could be acting as surrogates of traffic exhaust, which is a complex mixture of hundreds of toxic components (Kim et al. 2008; Singer et al. 2004). There is evidence that UFP number concentrations may be a more appropriate metric than gas or particle mass concentrations when evaluating health risk from traffic-related air pollution (Oberdörster et al. 2005). UFPs may be causal agents for the observed health effects due to their high pulmonary deposition efficiency, and their orders of magnitude higher number concentration and surface area that allows them to carry larger concentrations of adsorbed or condensed toxic air pollutants (e.g., oxidant gases, PAHs, and transition metals) to the fetus and the placenta (Oberdörster et al. 2005). UFPs contain a significant amount of PAHs, which have been linked to various measures of intrauterine growth retardation in studies in New York State (Choi et al. 2006, 2008; Perera et al. 2003, 2004), Krakow, Poland (Choi et al. 2006), and industrial areas of the Czech Republic (Dejmek et al. 2000). Yet, little is known to date about the etiologic role that UFPs and PAHs may play for preterm birth and preeclampsia.

Several hypotheses have been postulated to explain how air pollution may trigger PTD. Toxic compounds in traffic-generated air pollutants may interfere with placental development and subsequent nutrient and oxygen delivery to the fetus (Dejmek et al. 2000). Another potential mechanism of developmental toxicity is through the activation of the oxidative stress pathway. PTD may be triggered by an abnormal production or an early activation of cytokines favoring inflammation, even though increasing concentrations of inflammatory cytokines may be part of the body’s preparation for normal parturition (Engel et al. 2005; Keelan et al. 2003).

The mechanisms that initiate preeclampsia in pregnant women have been elusive (Mutter and Karumanchi 2008; Shah 2007). Pathology studies show that an abnormal development of an ischemic placenta with a high-resistance vasculature contributes to the development of preeclampsia. Endothelial dysfunction plays a central role in the pathogenesis of the syndrome. Multiple interconnected pathways linked to endothelial dysfunction involve oxidative stress, cytokine release, and a generalized intravascular inflammatory response (Baumwell and Karumanchi 2007). Exposure to traffic-related pollutants, such as UFPs and PAHs, can cause oxidative stress (Li et al. 2003; Nel et al. 2001; Oberdörster et al. 2005) and endothelial dysfunction (Tornqvist et al. 2007). Such exposure could thus contribute to the cardiovascular complications of preeclampsia as well as PTD (Gitto et al. 2002).

## Conclusions

Exposures to local traffic-generated air pollution modeled with CALINE4 for the entire pregnancy elevated the risk of preterm birth and preeclampsia in Southern California women. A 42% increased risk of preeclampsia was observed for the highest quartile of modeled traffic-related PM_2.5_ exposure during the entire pregnancy. For preterm birth, the exposure–response relation was strongest for VPTD with potentially serious consequences for the newborn. These results provide further evidence that traffic-related air pollution is associated with adverse reproductive outcomes.

## Figures and Tables

**Table 1:** Descriptive statistics of infants and mothers in our study in south Los Angeles County and Orange County, California, from 1997 to 2006 (*n* = 81,186).

Variable	Measure
Mother’s age (mean ± SD)	30.0 ± 6.2
Mother’s race (%)	
African American	8.8
Asian	9.9
Hispanic	32.1
White	40.3
Other	8.9
Male infant (%)	51.6
Gestational age [weeks (mean ± SD)]	38.7 ± 2.1
Preeclampsia (%)	3.0
Mild preeclampsia	74.9
Severe preeclampsia	18.2
HELLP syndrome	6.9
Term birth [≥ 37 weeks (%)]	91.7
Spontaneous	78.3
PTD [< 37 weeks (%)]	8.3
Spontaneous	87.2
MPTD [< 35 weeks (%)]	3.4
VPTD [< 30 weeks (%)]	1.0
Pyelonephritis (%)	0.2
Diabetes (%)	5.4
First child (%)	81.5
Delivery mode (%)	
Vaginal	73.1
Cesarean	26.9
Previous preterm infant (%)	1.1
Prenatal care insurance (%)	
Private	67.6
Government-sponsored or self-pay	28.4
Unknown	4.0
Poverty (%)^a^	14.2

a The percentage of the population living below the poverty level based on U.S. Census block group data for the year 2000.

**Table 2:** Pollutant averages and Pearson’s correlation coefficients for pollutants by pregnancy period.

					Pearson’s correlation coefficients							
					Entire pregnancy		First trimester		Second trimester		Third trimester	
Trimester	Pollutant	Mean^a^	IQR	SD	NO_x_	PM_2.5_	NO_x_	PM_2.5_	NO_x_	PM_2.5_	NO_x_	PM_2.5_
Entire pregnancy	NO_x_	7.23	5.65	5.22	1.00							
	PM_2.5_	1.82	1.35	1.33	0.90	1.00						
First trimester	NO_x_	7.45	6.17	5.68	0.91	0.83	1.00					
	PM_2.5_	1.83	1.44	1.37	0.84	0.94	0.91	1.00				
Second trimester	NO_x_	7.29	6.02	5.57	0.97	0.87	0.85	0.79	1.00			
	PM_2.5_	1.83	1.42	1.36	0.89	0.98	0.80	0.91	0.91	1.00		
Third trimester	NO_x_	7.14	5.88	5.54	0.91	0.83	0.71	0.68	0.85	0.79	1.00	
	PM_2.5_	1.84	1.43	1.39	0.85	0.95	0.70	0.82	0.79	0.90	0.91	1.00

a Units are parts per billion for NO_x_ and micrograms per cubic meter for PM2.5.

**Table 3:** Crude and adjusted ORs per IQR increase^a^ in traffic-related air pollutions for preeclampsia and preterm, moderate preterm, and very preterm birth.

Condition	No. of cases	Pollutant	Crude IOR (95% CI)	Adjusted^b^ IOR (95% CI)
Preeclampsia	2,442	NO_x_	1.15 (1.10–1.19)	1.11 (1.06–1.16)
		PM_2.5_	1.13 (1.09–1.17)	1.11 (1.06–1.15)
PTD (< 37 weeks)	6,712	NO_x_	1.12 (1.09–1.15)	1.06 (1.03–1.09)
		PM_2.5_	1.09 (1.06–1.11)	1.03 (1.01–1.06)
MPTD (< 35 weeks)	2,749	NO_x_	1.22 (1.18–1.26)	1.13 (1.09–1.18)
		PM_2.5_	1.15 (1.11–1.19)	1.07 (1.03–1.12)
VPTD (< 30 weeks)	775	NO_x_	1.32 (1.25–1.41)	1.25 (1.17–1.33)
		PM_2.5_	1.23 (1.16–1.31)	1.18 (1.10–1.26)

a Based on entire-pregnancy exposure. IQR was 5.65 ppb for NO_x_ and 1.35 μg/m^3^ for PM2.5.

b Adjusted for maternal age, maternal race/ethnicity, parity, prenatal care insurance type, poverty, and season of conception in all models. Additionally adjusted for pyelonephritis in PTD, MPTD, and VPTD models, and for diabetes in preeclampsia models.

## References

[b1-ehp-117-1773] Adar SD, Kaufman JD (2007). Cardiovascular disease and air pollutants: evaluating and improving epidemiological data implicating traffic exposure. Inhal Toxicol.

[b2-ehp-117-1773] Ahluwalia IB, Grummer-Strawn L, Scanlon KS (1997). Exposure to environmental tobacco smoke and birth outcome: increased effects on pregnant women aged 30 years or older. Am J Epidemiol.

[b3-ehp-117-1773] Ananth CV, Savitz DA, Luther ER, Bowes WA (1997). Preeclampsia and preterm birth subtypes in Nova Scotia. 1986 to 1992. Am J Perinatol.

[b4-ehp-117-1773] Baumwell S, Karumanchi SA (2007). Preeclampsia: clinical manifestations and molecular mechanisms. Nephron Clin Pract.

[b5-ehp-117-1773] Benson P (1989). CALINE4: A Dispersion Model for Predicting Air Pollutant Concentrations near Roadways.

[b6-ehp-117-1773] Benson PE (1992). A review of the development and application of the Caline3 and Caline4 models. Atmos Environ B Urban Atmos.

[b7-ehp-117-1773] Beverly O (2005). A Transportation Vision for Our Ports. Los Angeles Business Journal.

[b8-ehp-117-1773] Brauer M, Lencar C, Tamburic L, Koehoorn M, Demers P, Karr C (2008). A cohort study of traffic-related air pollution impacts on birth outcomes. Environ Health Perspect.

[b9-ehp-117-1773] Broderick BM, Budd U, Misstear BD, Ceburnis D, Jennings SG (2005). Validation of CALINE4 modelling for carbon monoxide concentrations under free-flowing and congested traffic conditions in Ireland. Int J Environ Pollut.

[b10-ehp-117-1773] California Air Resources Board (2008). EMFAC2007 version 2.30. Calculating Emissions Inventories for Vehicles in California, User’s Guide.

[b11-ehp-117-1773] California Environmental Protection Agency (2006). Diesel Particulate Matter Exposure Assessment Study for the Ports of Los Angeles and Long Beach.

[b12-ehp-117-1773] Challis JR, Lye SJ, Gibb W, Whittle W, Patel F, Alfaidy N (2001). Understanding preterm labor. Ann NY Acad Sci.

[b13-ehp-117-1773] Choi H, Jedrychowski W, Spengler J, Camann DE, Whyatt RM, Rauh V (2006). International studies of prenatal exposure to polycyclic aromatic hydrocarbons and fetal growth. Environ Health Perspect.

[b14-ehp-117-1773] Choi H, Rauh V, Garfinkel R, Tu Y, Perera FP (2008). Prenatal exposure to airborne polycyclic aromatic hydrocarbons and risk of intrauterine growth restriction. Environ Health Perspect.

[b15-ehp-117-1773] Chung JH, Garite TJ, Kirk AM, Hollard AL, Wing DA, Lagrew DC (2006). Intrinsic racial differences in the risk of cesarean delivery are not explained by differences in caregivers or hospital site of delivery. Am J Obstet Gynecol.

[b16-ehp-117-1773] Croes BE, Fujita EM (2003). Overview of the 1997 Southern California Ozone Study (SCOS97-NARSTO). Atmos Environ.

[b17-ehp-117-1773] Dejmek J, Solansky I, Beneš I, Lenicek J, Šrám RJ (2000). The impact of polycyclic aromatic hydrocarbons and fine particles on pregnancy outcome. Environ Health Perspect.

[b18-ehp-117-1773] de Kok TMCM, Driece HAL, Hogervorst JGF, Briede JJ (2006). Toxicological assessment of ambient and traffic-related particulate matter: a review of recent studies. Mutat Res.

[b19-ehp-117-1773] Delfino RJ (2002). Epidemiologic evidence for asthma and exposure to air toxics: linkages between occupational, indoor, and community air pollution research. Environ Health Perspect.

[b20-ehp-117-1773] Dietz PM, England LJ, Callaghan WM, Pearl M, Wier ML, Kharrazi M (2007). A comparison of LMP-based and ultrasound-based estimates of gestational age using linked California livebirth and prenatal screening records. Paediatr Perinat Epidemiol.

[b21-ehp-117-1773] Doyle LW (1995). Outcome to five years of age of children born at 24–26 weeks’ gestational age in Victoria. The Victorian Infant Collaborative Study Group. Med J Aust.

[b22-ehp-117-1773] Doyle LW (2008). Cardiopulmonary outcomes of extreme prematurity. Semin Perinatol.

[b23-ehp-117-1773] Duley L (1992). Maternal mortality associated with hypertensive disorders of pregnancy in Africa, Asia, Latin America and the Caribbean. Br J Obstet Gynaecol.

[b24-ehp-117-1773] Energy Information Administration (2008). International Annual Energy Outlook—Highlights. (DOE/EIA-0484).

[b25-ehp-117-1773] Engel SA, Erichsen HC, Savitz DA, Thorp J, Chanock SJ, Olshan AF (2005). Risk of spontaneous preterm birth is associated with common proinflammatory cytokine polymorphisms. Epidemiology.

[b26-ehp-117-1773] Fell DB, Dodds L, King WD (2004). Residential mobility during pregnancy. Paediatr Perinat Epidemiol.

[b27-ehp-117-1773] Fitz DR, Bufalino C (2002). Measurement of PM_10_ emission factors from paved roads using on-board particle sensors. http://www.epa.gov/ttn/chief/conference/ei11/dust/fitz.pdf.

[b28-ehp-117-1773] Gauderman WJ, Avol E, Lurmann F, Kuenzli N, Gilliland F, Peters J (2005). Childhood asthma and exposure to traffic and nitrogen dioxide. Epidemiology.

[b29-ehp-117-1773] Gitto E, Reiter RJ, Karbownik M, Tan DX, Gitto P, Barberi S (2002). Causes of oxidative stress in the pre- and perinatal period. Biol Neonate.

[b30-ehp-117-1773] Goldenberg RL, Culhane JF, Iams JD, Romero R (2008). Epidemiology and causes of preterm birth. Lancet.

[b31-ehp-117-1773] Gramotnev G, Brown R, Ristovski Z, Hitchins J, Morawska L (2003). Determination of average emission factors for vehicles on a busy road. Atmos Environ.

[b32-ehp-117-1773] Hamilton BE, Martin JA, Ventura SJ (2006). Births: Preliminary Data for 2005. Natl Vital Stat Rep.

[b33-ehp-117-1773] Heinrich J, Wichmann HE (2004). Traffic related pollutants in Europe and their effect on allergic disease. Curr Opin Allergy Clin Immunol.

[b34-ehp-117-1773] Hitchins J, Morawska L, Wolff R, Gilbert D (2000). Concentrations of submicrometre particles from vehicle emissions near a major road. Atmos Environ.

[b35-ehp-117-1773] Hoek G, Beelen R, de Hoogh K, Vienneau D, Gulliver J, Fischer P (2008). A review of land-use regression models to assess spatial variation of outdoor air pollution. Atmos Environ.

[b36-ehp-117-1773] Jerrett M (2006). Spatial Exposure Models for Assessing the Relation between Air Pollution and Childhood Asthma at the Intra-urban Scale. Progress Report EPA STAR Grant: RD-83184501-0.

[b37-ehp-117-1773] Jerrett M, Arain A, Kanaroglou P, Beckerman B, Potoglou D, Sahsuvaroglu T (2005). A review and evaluation of intraurban air pollution exposure models. J Expo Anal Environ Epidemiol.

[b38-ehp-117-1773] Keelan JA, Blumenstein M, Helliwell RJ, Sato TA, Marvin KW, Mitchell MD (2003). Cytokines, prostaglandins and parturition—a review. Placenta.

[b39-ehp-117-1773] Kim JJ, Huen K, Adams S, Smorodinsky S, Hoats A, Malig B (2008). Residential traffic and children’s respiratory health. Environ Health Perspect.

[b40-ehp-117-1773] Kline J (1989). Conception to Birth: Epidemiology of Prenatal Development.

[b41-ehp-117-1773] Levitin J, Harkonen J, Kukkonen J, Nikmo J (2005). Evaluation of the CALINE4 and CAR-FMI models against measurements near a major road. Atmos Environ.

[b42-ehp-117-1773] Li N, Sioutas C, Cho A, Schmitz D, Misra C, Sempf J (2003). Ultrafine particulate pollutants induce oxidative stress and mitochondrial damage. Environ Health Perspect.

[b43-ehp-117-1773] Lipfert FW, Wyzga RE (1999). Statistical considerations in determining the health significance of constituents of airborne particulate matter. J Air Waste Manag Assoc.

[b44-ehp-117-1773] Lynch CD, Zhang J (2007). The research implications of the selection of a gestational age estimation method. Paediatr Perinat Epidemiol.

[b45-ehp-117-1773] Maisonet M, Correa A, Misra D, Jaakkola JJ (2004). A review of the literature on the effects of ambient air pollution on fetal growth. Environ Res.

[b46-ehp-117-1773] Marmur A, Mamane Y (2003). Comparison and evaluation of several mobile source and line-source models in Israel. Transp Res D Transp Environ.

[b47-ehp-117-1773] Maroziene L, Grazuleviciene R (2002). Maternal exposure to low-level air pollution and pregnancy outcomes: a population-based study. Environ Health.

[b48-ehp-117-1773] Meis PJ, Goldenberg RL, Mercer BM, Iams JD, Moawad AH, Miodovnik M (1998). The preterm prediction study: risk factors for indicated preterm births. Maternal-Fetal Medicine Units Network of the National Institute of Child Health and Human Development. Am J Obstet Gynecol.

[b49-ehp-117-1773] Mone SM, Gillman MW, Miller TL, Herman EH, Lipshultz SE (2004). Effects of environmental exposures on the cardiovascular system: prenatal period through adolescence. Pediatrics.

[b50-ehp-117-1773] Mutter WP, Karumanchi SA (2008). Molecular mechanisms of preeclampsia. Microvasc Res.

[b51-ehp-117-1773] National Climatic Data Center (2008). Local Climatological Data.

[b52-ehp-117-1773] Nel AE, Diaz-Sanchez D, Li N (2001). The role of particulate pollutants in pulmonary inflammation and asthma: evidence for the involvement of organic chemicals and oxidative stress. Curr Opin Pulm Med.

[b53-ehp-117-1773] Oberdörster G, Oberdörster E, Oberdörster J (2005). Nanotoxicology: an emerging discipline evolving from studies of ultrafine particles. Environ Health Perspect.

[b54-ehp-117-1773] Olsen J, Basso O, Ahrens W, Pigeot I (2005). Reproductive epidemiology. Handbook of Epidemiology.

[b55-ehp-117-1773] Perera FP, Rauh V, Tsai WY, Kinney P, Camann D, Barr D (2003). Effects of transplacental exposure to environmental pollutants on birth outcomes in a multiethnic population. Environ Health Perspect.

[b56-ehp-117-1773] Perera FP, Rauh V, Whyatt RM, Tsai WY, Bernert JT, Tu YH (2004). Molecular evidence of an interaction between prenatal environmental exposures and birth outcomes in a multiethnic population. Environ Health Perspect.

[b57-ehp-117-1773] Pinkerton KE, Joad JP (2006). Influence of air pollution on respiratory health during perinatal development. Clin Exp Pharmacol Physiol.

[b58-ehp-117-1773] Ponce NA, Hoggatt KJ, Wilhelm M, Ritz B (2005). Preterm birth: the interaction of traffic-related air pollution with economic hardship in Los Angeles neighborhoods. Am J Epidemiol.

[b59-ehp-117-1773] Ritz B, Wilhelm M, Hoggatt KJ, Ghosh JK (2007). Ambient air pollution and preterm birth in the environment and pregnancy outcomes study at the University of California, Los Angeles. Am J Epidemiol.

[b60-ehp-117-1773] Ritz B, Yu F, Chapa G, Fruin S (2000). Effect of air pollution on preterm birth among children born in Southern California between 1989 and 1993. Epidemiology.

[b61-ehp-117-1773] Rudra C, Williams M (2006). A prospective study of periconceptional ambient air pollutant exposures and preeclampsia risk. Epidemiology.

[b62-ehp-117-1773] Sarnat JA, Holguin F (2007). Asthma and air quality. Curr Opin Pulm Med.

[b63-ehp-117-1773] Shah DM (2007). Preeclampsia: new insights. Curr Opin Nephrol Hypertens.

[b64-ehp-117-1773] Sibai B, Dekker G, Kupferminc M (2005). Preeclampsia. Lancet.

[b65-ehp-117-1773] Singer BC, Hodgson AT, Hotchi T, Kim JJ (2004). Passive measurement of nitrogen oxides to assess traffic-related pollutant exposure for the East Bay Children’s Respiratory Health Study. Atmos Environ.

[b66-ehp-117-1773] Slama R, Morgenstern V, Cyrys J, Zutavern A, Herbarth O, Wichmann HE (2007). Traffic-related atmospheric pollutants levels during pregnancy and offspring’s term birth weight: a study relying on a land-use regression exposure model. Environ Health Perspect.

[b67-ehp-117-1773] Šrám RJ, Binková BB, Dejmek J, Bobak M (2005). Ambient air pollution and pregnancy outcomes: a review of the literature. Environ Health Perspect.

[b68-ehp-117-1773] Tornqvist H, Mills NL, Gonzalez M, Miller MR, Robinson SD, Megson IL (2007). Persistent endothelial dysfunction in humans after diesel exhaust inhalation. Am J Respir Crit Care Med.

[b69-ehp-117-1773] U.S. Census Bureau (2004). 2000 Census of Population and Housing. Summary Tape File 3A.

[b70-ehp-117-1773] Wilhelm M, Ritz B (2003). Residential proximity to traffic and adverse birth outcomes in Los Angeles County, California, 1994–1996. Environ Health Perspect.

[b71-ehp-117-1773] Wilhelm M, Ritz B (2005). Local variations in CO and particulate air pollution and adverse birth outcomes in Los Angeles County, California, USA. Environ Health Perspect.

[b72-ehp-117-1773] Windham GC, Hopkins B, Fenster L, Swan SH (2000). Prenatal active or passive tobacco smoke exposure and the risk of preterm delivery or low birth weight. Epidemiology.

[b73-ehp-117-1773] Woodruff T, Morello-Frosch R, Jesdale B (2008). Air pollution and preeclampsia among pregnant women in California, 1996–2004 [Abstract]. Epidemiology.

[b74-ehp-117-1773] Wu J, Houston D, Lurmann F, Ong P, Winer A (2009). Exposure of PM_2.5_ and EC from diesel and gasoline vehicles in communities near the Ports of Los Angeles and Long Beach, California. Atmos Environ.

[b75-ehp-117-1773] Wu J, Lurmann F, Winer A, Lu R, Turco R, Funk T (2005). Development of an individual exposure model for application to the Southern California Children’s Health Study. Atmos Environ.

[b76-ehp-117-1773] Yang CY, Chang CC, Chuang HY, Ho CK, Wu TN, Tsai SS (2003). Evidence for increased risks of preterm delivery in a population residing near a freeway in Taiwan. Arch Environ Health.

[b77-ehp-117-1773] Zhu YF, Hinds WC, Kim S, Shen S, Sioutas C (2002). Study of ultrafine particles near a major highway with heavy-duty diesel traffic. Atmos Environ.

